# Multi-Omics Exploration of the Mechanism of Curcumol to Reduce Invasion and Metastasis of Nasopharyngeal Carcinoma by Inhibiting NCL/EBNA1-Mediated UBE2C Upregulation

**DOI:** 10.3390/biom14091142

**Published:** 2024-09-09

**Authors:** Haiping Liu, Juan Wang, Lin Wang, Wei Tang, Xinyue Hou, Yi Zhun Zhu, Xu Chen

**Affiliations:** 1 School of Pharmacy, Faculty of Medicine, Macau University of Science and Technology, Macau 999078, China; lhp1204@163.com (H.L.); wl9700@163.com (L.W.); tang678998@163.com (W.T.); 2 Pharmacology Laboratory of Prevention and Treatment of High Incidence of Disease, Guilin Medical University, Guilin 541199, China; juanlovelife@163.com (J.W.); 13237830549@163.com (X.H.)

**Keywords:** curcumol, transcriptomics, proteomics, metabolomics, nasopharyngeal carcinoma, NCL, EBNA1, UBE2C

## Abstract

Nasopharyngeal carcinoma (NPC) is closely linked to Epstein–Barr virus (EBV) infection. *Curcumae Rhizoma*, a traditional Chinese herb, has shown antitumor effects, primarily through its component curcumol (Cur), which has been shown to reduce NPC cell invasion and migration by targeting nucleolin (NCL) and Epstein–Barr Virus Nuclear Antigen 1 (EBNA1). We constructed an EBV-positive NPC cell model using C666-1 cells and performed transcriptomics studies after treatment with curcumol, which revealed a significant enrichment of ubiquitin-mediated proteolysis, the PI3K-AKT and mTOR signaling pathways, cell cycle and apoptosis involved in tumor invasion and migration. To investigate the importance of NCL and EBNA1 in curcumol-resistant EBV-positive NPC, we performed a multi-omics study using short hairpin NCL (shNCL) and shEBNA1 EBV-positive NPC cells, and the proteomics results showed enrichment in complement and coagulation cascades and ubiquitin-mediated proteolysis signaling pathways. Here, we focused on ubiquitin-conjugating enzyme E2C (UBE2C), which plays an important role in the ubiquitin-mediated proteolysis signaling pathway. In addition, metabolomics revealed that UBE2C is highly associated with 4-Aminobutanoic acid (GABA). In vitro studies further validated the function of the key targets, suggesting that UBE2C plays an important role in NCL and EBNA1-mediated curcumol resistance to nasopharyngeal carcinoma invasion and metastasis.

## 1. Introduction

The global incidence of nasopharyngeal carcinoma (NPC), as a malignant tumor affecting the head and neck, cannot be ignored. According to the International Agency for Research on Cancer, the number of new NPC cases worldwide in 2018 was approximately 129,000 cases [1]. What is particularly remarkable is that more than 70% of these new cases are concentrated in East and Southeast Asia. The development of NPC is a multifactorial and complex process, in which Epstein–Barr virus (EBV) infection is regarded as one of the key risk factors. EBV is a herpes gamma virus that infects more than 90% of the world’s population, but in the vast majority of carriers, EBV does not cause unusual symptoms. In southern China, EBV-positive infections are the most important risk factor for the occurrence and development of NPC, accounting for more than 95% of cases, and invasion and distant migration are the main causes of recurrence and death in patients with EBV-positive NPC [1,2,3]. In addition, the genetic and environmental factors of the individual host should not be ignored [4,5,6]. Despite continuous advances in NPC diagnostic and treatment technologies and improved patient survival rates, patients with advanced stages of the disease often face high recurrence rates and poor prognosis due to invasive and distant metastases, and more than 70% of the patients are already in advanced stages at the time of diagnosis [7]. Epstein–Barr Virus Nuclear Antigen 1 (EBNA1), as an essential viral protein for the stabilization of EBV infection, is widely expressed in NPC tissues [4]. In EBV-positive NPC patients, invasion and migration are the main causes of recurrence and death; so, their inhibition is essential for optimal treatment.

The current treatment of NPC is radiotherapy combined with chemotherapy, but the incidence of drug resistance and adverse reactions is high [8]. We previously reported that curcumol (Cur), an effective component extracted from the oil of *Curcumae Rhizoma*, has significant anti-NPC, colorectal cancer, and colon cancer activity [9,10]. It effectively inhibits the invasion and metastasis of NPC cells while inducing cell autophagy [11], cycle arrest, and apoptosis processes [12]. We have previously identified NCL as one of the target proteins of curcumol against NPC invasion and metastasis [13]. EBNA1 could interact with NCL and curcumol inhibited the invasion and metastasis of NPC cells by targeting NCL to inhibit EBNA1 [10]. Therefore, NCL and EBNA1 played an important role in curcumol anti-NPC, and more and deeper mechanisms behind them need to be explored.

This study aimed to reveal the underlying mechanism of curcumol’s anti-NPC effect mediated by NCL/EBNA1 using multi-omics. Applying diversified collective technologies and advanced methods, the ‘Omic’ science analyses the complex molecules and specific genes in living organisms, revealing the deep-seated mechanisms and laws behind the phenomena of life [14]. The combination of multi-omics helped us focus on the expression coherence of some important proteins from the RNA level to the protein level [15,16,17]. Especially given we know curcumol has a good activity of NPC, based on using the transcriptomics analysis study out the differentially expressed genes (DEGs), differentially expressed proteins (DEPs) were carried out by the proteomics analysis with knockdown of the key proteins of NCL and EBNA1, respectively, and metabolomics analysis of the correlation of differential metabolites with key proteins. The above DEGs and DEPs were analyzed jointly. This way, the key factors and the mechanism of action of the drug can be better identified.

This study aimed to elucidate the underlying mechanism of NCL/ EBNA1-mediated curcumol anti-NPC by multi-omics methods. The data showed that curcumol inhibits NPC invasion and metastasis in association with the ubiquitin-conjugating enzyme E2C (UBE2C), a potential target for anti-EBV-positive NPC therapy. It also provides a new perspective for studying the mechanism of small-molecule drugs derived from natural plants.

## 2. Materials and Methods

### 2.1. Materials

Curcumol (purity ≥ 98%) was purchased from the Guizhou Di Technology Co., Ltd. (Guiyang, China) (product No.: C100409-211024, CAS No.: 4871-97-0). It was dissolved in anhydrous ethanol as a mother solution. Other solutions and antibodies used are as follows: DMEM (Gibco, Grand Island, NE, USA); FBS (Gibco, Grand Island, NE, USA); penicillin-streptomycin (Solarbio, Beijing, China); RIPA lysis buffer (Beyotime Biotechnology, Shanghai, China); BCA protein assay kit (Beyotime Biotechnology, Shanghai, China); Antibody against β-actin (ZSGB-BIO, Beijing, China); NCL (Abcam, Cambridge, UK); EBNA1 (Santa Cruz Biotechnology, Santa Cruz, CA, USA); and UBE2C (P22620, ProMab, Richmond, CA, USA).

### 2.2. Animal Experiment Ethics

Ethical approval for the use of animals was obtained before the start of this study from the Institutional Animal Care and Use Committee of Guilin Medical University (Guilin, China) (qualification certificate No.: (Gui) 2020-0005).

### 2.3. Cell Culture

The C666-1 cells were derived from Xiangya Hospital (Changsha, China) and were cultured in DMEM medium containing 10% Fetal Bovine Serum (FBS) and a 1% penicillin–streptomycin mixture in a cell culture incubator at a constant temperature of 37 °C with 5% CO_2_. In addition, to ensure the purity and health of the cell line, a comprehensive mycoplasma contamination test was performed every 2 to 3 months.

### 2.4. Cell Transfection

The shNCL plasmid for NCL knockdown, shEBNA1 plasmid for EBNA1 knockdown, and con077, con634, and con660 plasmids were purchased from Shanghai Gene Chemical Co., Ltd. (Shanghai, China). C666-1 cells were seeded into 6-well plates and transfected with Lipofectamine 3000 when the density reached 70%. After 6 h, a fresh DMEM medium containing 10% FBS was replaced for several consecutive days. Stable transfected cell lines were screened using puromycin.

### 2.5. Transcriptomics Analysis

NPC cells were treated with curcumol and transcriptomics analysis was performed to further investigate the inhibitory mechanism of curcumol on NPC. Transcriptomics analysis mainly focused on the changes in mRNA abundance in NPC cells with the curcumol treatment group and control group. Trizol reagent (Thermo Fisher Scientific, Waltham, MA, USA) was applied to extract total RNA. The constructed libraries were qualified by Agilent 2100 Bioanalyzer and sequenced using Illumina sequencer (Illumine, San Diego, CA, USA). Library construction, sequencing, and original data processing were carried out by OE Biotech Co., Ltd. (Shanghai, China). DESeq and |log2FoldChange| ≥ 1 and *p* < 0.05 were used to identify DEGs.

### 2.6. Data Independent Acquisition (DIA) Quantitative Proteomics Analysis

Proteomics analysis was used to explore the mechanism of NCL and EBNA1-mediated suppression of NPC. NPC cell samples were divided into 3 groups: the shNCL treatment group, the shEBNA1 treatment group, and the control group (C666-1 con077). Each group consisted of three samples. Appropriate amounts of SDT lysis solution were added to each sample and transferred to EP tubes. Extracted proteins were temporarily stored at −80 °C and transported on dry ice to Shanghai Bioprofile Technology Co., Ltd. (Shanghai, China) for DIA quantitative proteomics mass spectrometry on a Q-Exactive HF-X (Thermo Scientific) mass spectrometer. Database for uniprot-Homo sapiens (Human) [9606]-207393-20221227. Fasta, from https://www.uniprot.org, accessed on 27 December 2022. The results of the DIA raw MS files were imported into Spectronaut (version 17, Biognosys AG, Zurich Switzerland) for direct DIA analysis. Significantly, DEPs were accepted as follows: |log2FoldChange| ≥ 1 and *p* < 0.05.

### 2.7. Metabolomics Data Analysis

In this study, metabolomics analysis was performed using a UPLC-ESI-Q-Orbitrap-MS system (UHPLC, Shimadzu Nexera X2 LC-30AD, Shimadzu, Japan), and each sample was filtered through disposable 0.22 µm cellulose acetate and separated on an ACQUITY UPLC^®^ HSS T3 column (2.1 × 100 mm, 1.8 μm) (Waters, Milford, MA, USA) with a gradient elution of the mobile phase. Mass spectrometry data acquisition was performed in positive and negative ion modes, and peak alignment, correction, and area extraction were processed by MS-DIAL and compared with the database to identify metabolites. Multivariate analysis was performed using R software (version 4.0.3) and PLS-DA and OPLS-DA models, and statistically significant differential metabolites were screened by VIP values and *t*-tests (VIP > 1.0, *p* < 0.05), and further clustering analyses were performed to reveal metabolic patterns.

### 2.8. Bioinformatic Analysis

Volcano plots were visualized for the data of DEGs and DEPs using the R language (v 3.2.0) and its ggplot2 R package. Gene ontology (GO) and Kyoto Encyclopedia of Genes and Genomes (KEGG) were performed using a hypergeometric distribution test, and the column diagrams and heatmaps of significantly enriched terms were further constructed.

### 2.9. Scratch Assay

The logarithmic growth phase cells were selected and inoculated into 6-well plates at 1 × 10^5^ cells/mL, with 3 parallel wells in each group. When the cells were spread to the bottom of the wells, a 200 μL sterile lance tip was used to scratch along the scale, and the culture was changed to a fresh complete medium after light washing with PBS. Photographs were taken with an inverted microscope at 0 and 24 h, and the wound healing rate was analyzed by ImageJ to assess the cell migration ability.

### 2.10. Transwell Assay

Matrigel was mixed with serum-free DMEM medium at a volume ratio of 1:8 and dissolved at 4 °C overnight. Afterward, this mixture was spread evenly on the bottom of the upper chamber of a pre-cooled transwell chamber to avoid air bubbles. Immediately after that, 200 μL suspension containing 1 × 10^4^ cells was injected into the upper chamber of the transwell chamber. An appropriate amount of complete culture medium was also added to the lower chamber as an inducer of cell migration. After 24 h of incubation, those cells that had migrated to the lower chamber were fixed using paraformaldehyde solution, and the fixed cells were subsequently stained using crystal violet solution. The migrated cells were observed and counted by an inverted microscope; this process was repeated 3 times, and the counts were averaged.

### 2.11. Quantitative Real-Time (qRT) PCR

We first extracted total RNA using the Trizol reagent and selected the ACTIN gene as an endogenous reference gene to normalize the expression of the target gene by its expression level. In quantifying the gene expression levels, we adopted the comparative threshold cycle method (ΔΔCt), and further used the formula 2^−ΔΔCT^ to estimate the relative expression levels of the target genes. To ensure the accuracy and reliability of the experimental results, we adopted a rigorous experimental design in which three independent experiments were conducted for each sample or condition. The primers used to amplify the target sequences are listed in Table 1.

### 2.12. Western Blot

C666-1 cells were placed in 7 cm Petri dishes and subsequently treated with 100 μg/mL of curcumol solution for a 24 h intervention. Upon completion of the treatment, the cell precipitate was collected, and immediately following the addition of an appropriate amount of RIPA lysate to the precipitate, lysis was carried out at a low temperature of 4 °C for 30 min. Upon completion of lysis, the lysate mixture was placed in a high-speed cryo-centrifuge and centrifuged at 12,000 rpm for 20 min at 4 °C to isolate the proteins in the supernatant. Protein quantification of the cell samples was performed using a BCA kit (Beyotime Biotechnology Co., Ltd., Shanghai, China). Subsequently, the quantified protein samples were subjected to SDS-PAGE electrophoresis to separate proteins of different molecular weights. After electrophoresis, the proteins were transferred from the gel to the NC membrane (Solarbio, Beijing, China) and subjected to closure to eliminate non-specific binding. After sealing, primary and secondary antibodies against the target proteins were sequentially added to the membrane for binding. Finally, the signal of secondary antibody binding was detected by adding an ECL reagent (Biosharp, Hefei, China). Western blot images were measured in grey scale values using Image Lab software (version 6.0.0 Build 25) and further statistically analyzed. In order to enhance the reliability and reproducibility of the experimental results, the experimental data were repeated 3 times, and the data from each experiment were recorded and statistically analyzed.

### 2.13. Experimental Model in Nude Mice

In this study, we strictly followed the guidelines and approvals of the experimental animal ethical committee of Guilin Medical University (certificate of approval No.: (Gui) 2020-0005), and used SPF-grade BALB/c nude mice, 4–5 weeks of age, weighing (17 ± 2) g, which were purchased from Hunan Innovation value business Co., Ltd. (Changsha, China) (qualification certificate No.: (Xiang) 2019-0004). For the experimental design, we selected three cell lines, shNCL C666-1 cells (Con634), shEBNA1 C666-1 cells (Con660), and their corresponding control cells (Con077). The cells were dissolved in 100 µL of PBS at a concentration of 3 × 10^5^ cells and injected separately into the axillary region of nude mice. Subsequently, the weight and tumor volume changes in the nude mice were monitored and recorded every three days. Once the tumor diameter exceeded the preset criterion of 4 mm, the mice were divided into two groups randomly: the experimental group and the control group. Mice in the experimental group received daily gavage treatment with curcumol (80 mg/kg), whereas an equal amount of saline was gavaged to the control mice to exclude non-specific effects. After 2 weeks of intervention, all mice were deeply anesthetized by intraperitoneal injection of 40 mg/kg of sodium pentobarbital and subsequently humanely executed. Subsequently, tumor tissue samples were fixed and collected in 4% paraformaldehyde solution. The samples were stored in a refrigerator at −80 °C for subsequent use in other relevant analyses.

### 2.14. Statistical Analysis

Statistical analysis was carried out by GraphPad Prism software (version 10.1.2). Comparisons between 2 groups were carried out using an unpaired Student’s *t*-test, and one-way ANOVA was used between multiple groups. *p* < 0.05 was regarded as statistically significant.

## 3. Results

### 3.1. Curcumol Treatment Altered the Transcriptomics Profile of EBV-Positive NPC Cells

**Figure 1:**
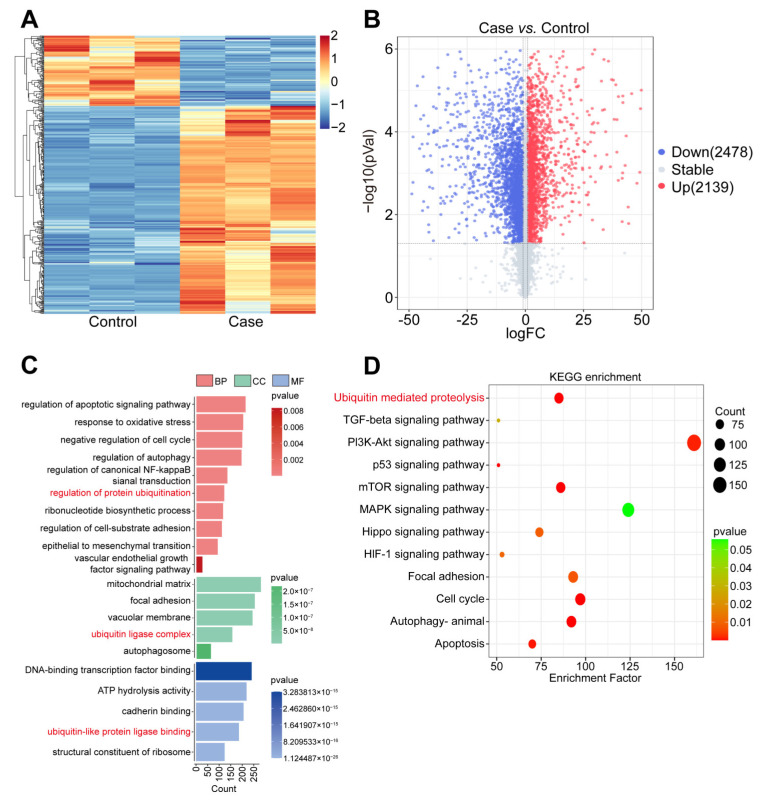
Curcumol treatment altered the transcriptomics profile of NPC cells. (**A**) Heatmap of DEGs after curcumol treatment. (**B**) Volcano plots of DEGs after curcumol treatment. (**C**) GO annotation of DEGs, which were divided into BP, CC, and MF. (**D**) KEGG pathways’ enrichment analysis bubble plot of DEGs.

From the statistics shown in the heatmap and volcano diagram analyses of DEGs (Figure 1A,B), a total of 2478 DEGs were down-regulated and 2139 DEGs were up-regulated after curcumol treatment compared with the control group. Further analyzed by GO annotation (Figure 1C), these DEGs were mainly enriched in several key biological processes (BP), including regulation of the apoptosis signaling pathway, response to oxidative stress, negative cell cycle, regulation of autophagy, regulation of canonical NF-κB signal transduction, and regulation of the protein ubiquitination process. Cellular component (CC) analyses indicated that DEGs were primarily engaged in the mitochondrial matrix, focal adhesion, vacuolar membrane, ubiquitin ligase complex, and autophagosome. In the molecular function (MF) category, DEGs were identified to be associated with DNA-binding transcription factor binding, ATP hydrolysis activity, cadherin binding, ubiquitin-like protein ligase binding, and the structural constituent of ribosome. Based on the KEGG-enriched pathway analyses, several pathways were involved in the invasion and migration of tumor cells after the treatment with curcumol compared to the control group, such as ubiquitin-mediated proteolysis, TGF-β, PI3K-Akt, p53, mTOR, MAPK, Hippo, and HIF-1 signaling pathway, focal adhesion, cell cycle, animal autophagy, and apoptosis (Figure 1D). Notably, the ubiquitin-mediated proteolysis, as a central mechanism, played a key role in all these regulatory processes, which, in turn, were involved in the invasion and migration process of tumor cells.

### 3.2. Knockdown of NCL or EBNA1 Altered the Proteomics Profile of EBV-Positive NPC Cells

**Figure 2:**
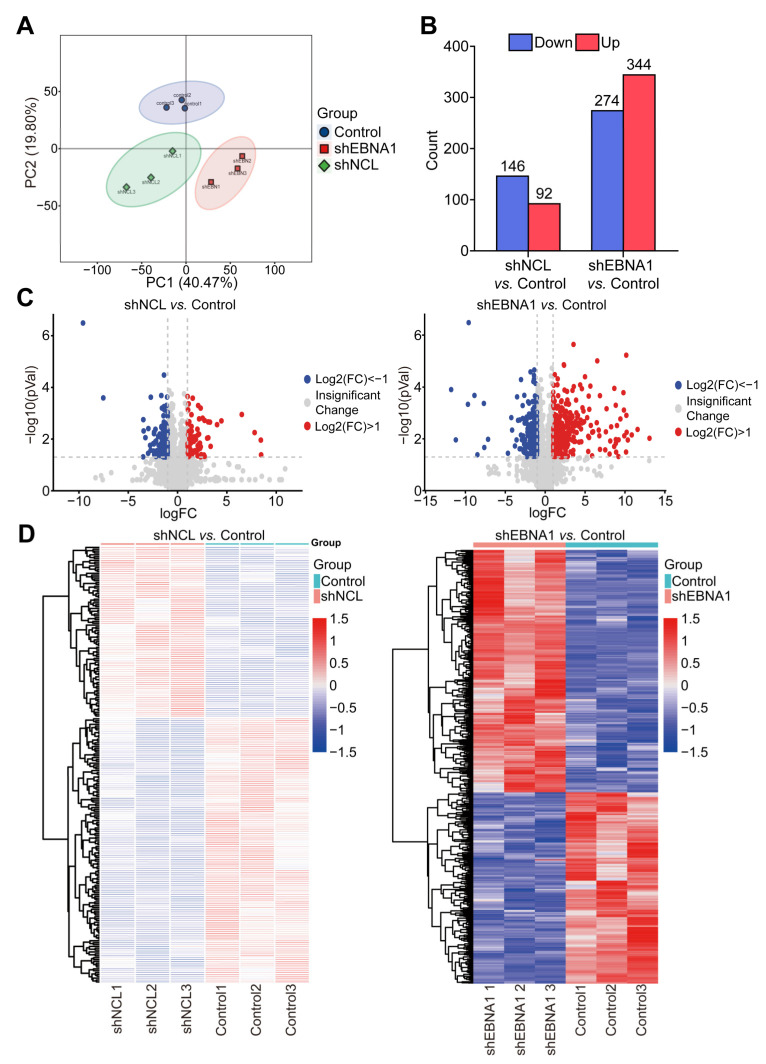
Proteomics analysis of DEPs in NPC cells of the shNCL or shEBNA1. (**A**) Principal component analysis (PCA) of the DEPs. (**B**) Up-regulation and down-regulation of the amount of protein. (**C**) Volcano plots of the enriched DEPs. (**D**) Heatmaps of the enriched DEPs.

Our previous study found that NCL and EBNA1 played crucial roles in the curcumol inhibition of NPC. To dig deeper into the key downstream proteins of this mechanism, NPC cells were treated with shNCL and shEBNA1, and the collection of cells was proteomically analyzed by DIA. The different groups were well separated (Figure 2A). Compared with the control group, there were 146 down-regulated proteins and 92 up-regulated proteins in the shNCL group. There were 274 down-regulated and 344 up-regulated proteins in the shEBNA1 group (Figure 2B). Volcano plots and corresponding heatmaps of DEPs also collectively revealed significant changes in protein expression levels (Figure 2C,D).

To further explore the specific effects of NCL and EBNA1 on the biological process of NPC, we performed GO analysis of the DEPs between the “shNCL vs. Control”, and the DEPs between the “shEBNA1 vs. Control”, respectively. This analysis covered the three dimensions of BP, CC, and MF. The administration of both shNCL or shEBNA1 mainly involves cellular process, metabolic process, positive regulation of the biological process, regulation of the biological process, biological regulation, and response to stimulus in terms of BP, cellular anatomical entity, and protein-containing complex in terms of CC, binding and catalytic activity in terms of MF (Supplementary Figure S1A,B).

**Figure 3:**
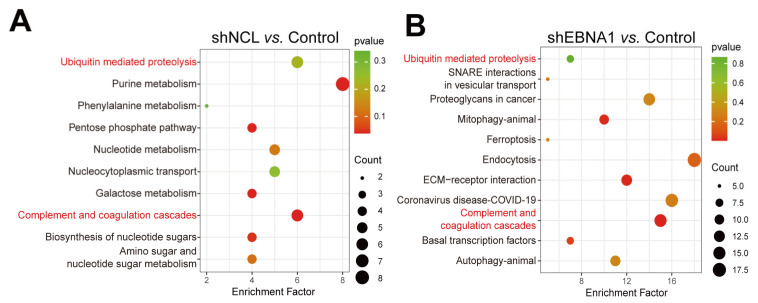
KEGG analysis of DEPs in NPC cells with shNCL or shEBNA1. (**A**) Significantly enriched KEGG pathways based on the DEPs in “shNCL vs. Control”. (**B**) Significantly enriched KEGG pathways based on the DEPs in “shEBNA1 vs. Control”.

The most highly enriched KEGG pathways were listed as “shNCL vs. Control” and “shEBNA1 vs. Control” (Figure 3A,B). Among these pathways, purine metabolism, pentose phosphate pathway, galactose metabolism, complement and coagulation cascades, biosynthesis of nucleotide sugars, amino sugar and nucleotide sugar metabolism, nucleotide metabolism, and ubiquitin-mediated proteolysis were mainly enriched in the shNCL group compared to the control group. Complement and coagulation cascades, ECM-receptor interaction, animal mitophagy, basal transcription factors, endocytosis, SNARE interactions in vesicular transport, animal autophagy, ferroptosis, and ubiquitin-mediated proteolysis were enriched in the shEBNA1 group compared to the control group. Taken together, ubiquitin-mediated proteolysis as well as complement and coagulation cascades were enriched in both sets of analyses. Interestingly, ubiquitin-mediated proteolysis was not only significantly enriched in transcriptomic analyses, but also showed a high degree of concordance at the proteomic level, strongly suggesting its centrality in cellular regulation.

**Figure 4:**
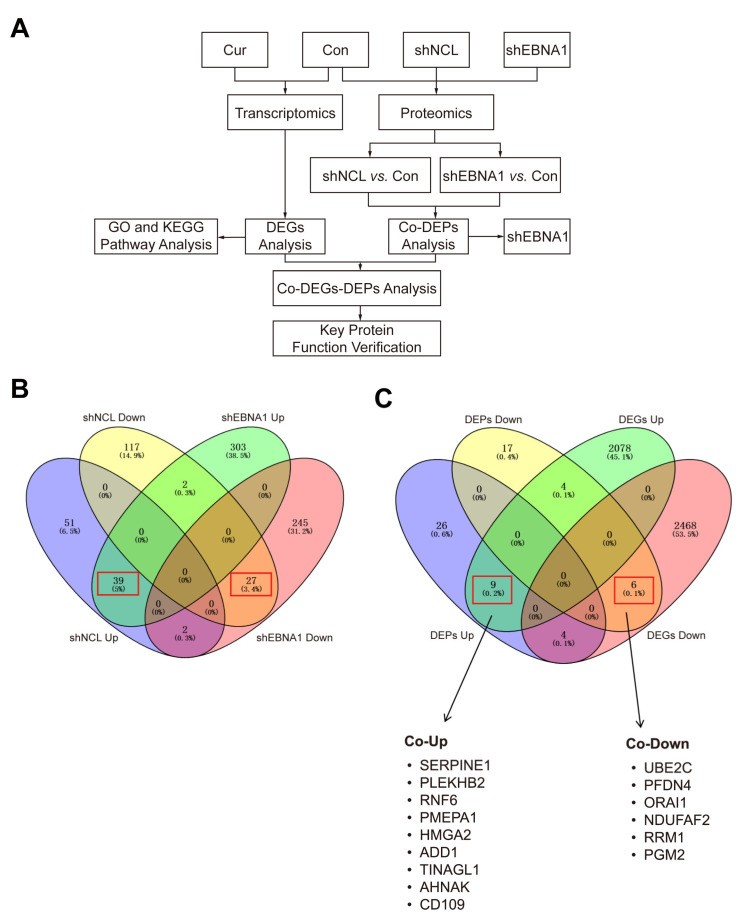
Correlation analysis of transcriptomics and proteomics revealed key proteins in the inhibition of NPC by curcumol. (**A**) Flow chart of key target approaches for curcumol inhibition of NPC. (**B**) Venn diagram of DEPs for “shNCL vs. Control” and “shEBNA1 vs. Control”, red boxes indicate the number of DEPs regulated in the same direction. (**C**) Venn diagram of DEGs and DEPs, and common key targets shown, red boxes were same with (**B**).

To find the key targets, we performed an association analysis of DEGs and common DEPs (Figure 4A). Given our previous findings that NCL and EBNA1 played a synergistic role in curcumol’s inhibitory effect on NPC invasion and migration, we performed a joint analysis of the proteomics data for “shNCL vs. Control” and “shEBNA1 vs. Control” with discriminate directions. The results indicated that a total of 39 up-regulated and 27 down-regulated proteins were obtained at the intersection of “shNCL vs. Control” and “shEBNA1 vs. Control” (Figure 4B). Subsequently, we performed an enrichment heatmap display of the 66 DEPs regulated in the same direction obtained by the Venn diagram (Supplementary Figure S2). The heatmaps showed the expression of each differentially expressed protein in each sample. The Venn diagram results of the 66 DEPs above showed that there were nine up-regulated targets, including SERPINE1, PLEKHB2, RNF6, PMEPA1, HMGA2, ADD1, TINAGL1, AHNAK and CD109, and six down-regulated targets, including UBE2C, PFDN4, ORAI1, NDUFAF2, RRM1, and PGM2 (Figure 4C).

**Figure 5:**
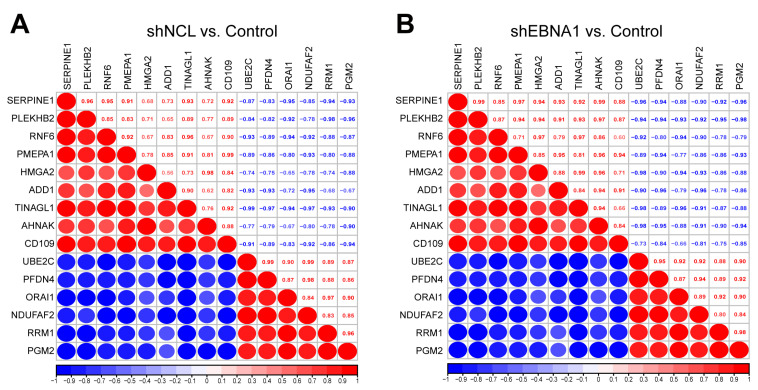
Correlation analysis of the key proteins. Red represented a positive correlation and blue represented a negative correlation. Larger red or blue circles indicated a stronger correlation. The correlation coefficients were shown on the diagonal box. (**A**) The group of “shNCL vs. Control”. (**B**) The group of “shEBNA1 vs. Control”.

Furthermore, we performed correlation analysis on the above 15 key targets. We found that there was a positive correlation between SERPINE1 and PLEKHB2, RNF6 and TINAGL1, HMGA2 and AHNAK, UBE2C and PFDN4, RRM1 and PGM2, with correlation coefficients all greater than 0.95 in two groups. Moreover, we identified a negative correlation between PLEKHB2 and RRM1, PLEKHB and PGM2, ADD1, and NDUFAF2, and TINAGL1 and UBE2C, with correlation coefficients all lower than −0.95 in two groups (Figure 5A,B). UBE2C and several other key proteins even reached |0.99| correlation coefficients. These data suggested the potential mechanism behind NCL/EBNA1-mediated inhibiting of NPC invasion and migration of the relationship between the key proteins.

### 3.3. Knockdown of NCL or EBNA1 Altered the Metabolomic Profile of EBV-Positive NPC Cells

**Figure 6:**
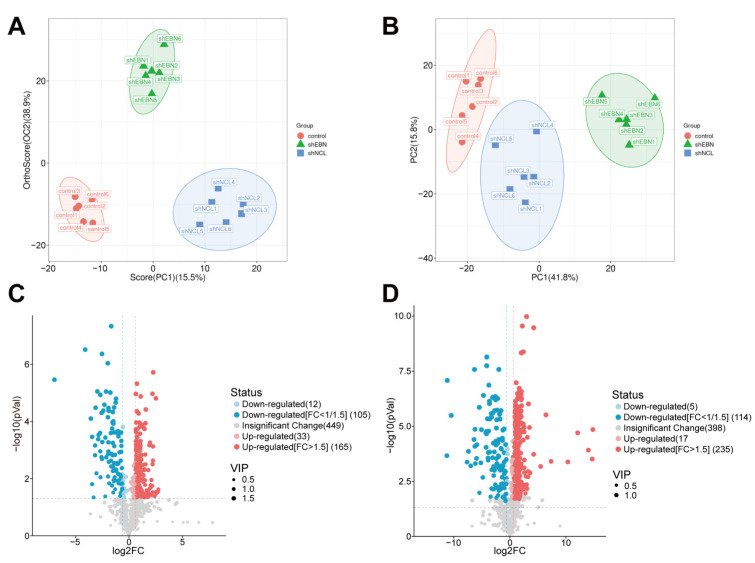
Knockdown of NCL or EBNA1 altered the metabolomic profile of EBV-positive NPC cells. (**A**) OPLS-DA analysis. (**B**) PCA analysis. (**C**) Volcano diagram of “shNCL vs. Control”. (**D**) Volcano diagram of “shEBNA1 vs. Control”.

We performed metabolomic analyses of NPC cells from Control, shNCL, and shEBNA1 groups. OPLS-DA (R2Y = 0.966, Q2 = 0.812) and PCA analyses showed small intra-group differences between the control and shNCL groups, and control and shEBNA1 groups, and large inter-group differences (Figure 6A,B). This result indicated differences in the metabolites between the different groups, which warranted further analysis. The major differential metabolisms in the shNCL and shEBNA1 groups compared to the control group included lipids and lipid-like molecules, organic acids and derivatives, organoheterocyclic compounds, benzenoids, phenylpropanoids and polyketides, and organic oxygen compounds (Supplementary Figures S3A and S4A). The results of the circular heatmap and volcano map showed that a total of 198 metabolites were up-regulated and 117 metabolites were down-regulated in the shNCL group compared with the control group (Figure 6C, Supplementary Figure S3B). In addition, a total of 252 metabolites were up-regulated and 119 metabolites were down-regulated in the shEBNA1 group (Figure 6D, Supplementary Figure S4B).

### 3.4. Combined Metabolomics and Proteomics Analyses Further Revealed That 4-Aminobutanoic Acid (GABA) Is Highly Negatively Correlated with UBE2C

Among the 15 key targets, we focused on a key target on the ubiquitin-mediated protein hydrolysis pathway, UBE2C. Based on this, we performed KEGG enrichment of differential metabolites and selected pathways closely related to cancer and amino acid metabolism for subsequent analysis. There were 21 relevant pathways screened from the “shNCL vs. Control”, and 22 relevant pathways screened from the “shEBNA1 vs. Control”, and they were highly overlapped (Supplementary Figure S5A,B). Subsequently, we listed the relevant metabolic pathways and their corresponding differential metabolites, and the results showed that one metabolite participated in multiple signaling pathways. These metabolic pathways mainly included protein digestion and absorption, central carbon metabolism in cancer, biosynthesis of amino acids, Aminoacyl-tRNA biosynthesis, arginine and proline metabolism, phenylalanine metabolism, valine, leucine and isoleucine biosynthesis, tryptophan metabolism, and mTOR signaling pathway, etc. Differential metabolites mainly included DL-Arginine, DL-Glutamine, Polyphenylalanine, DL-Valine, 4-Aminobutanoic acid, Estradiol, alpha-Ketoisocaproic acid, Uracil, Phenylacetylglycine, Glycocyamine and p-Coumaric acid (Supplementary Figure S6A,B).

**Figure 7:**
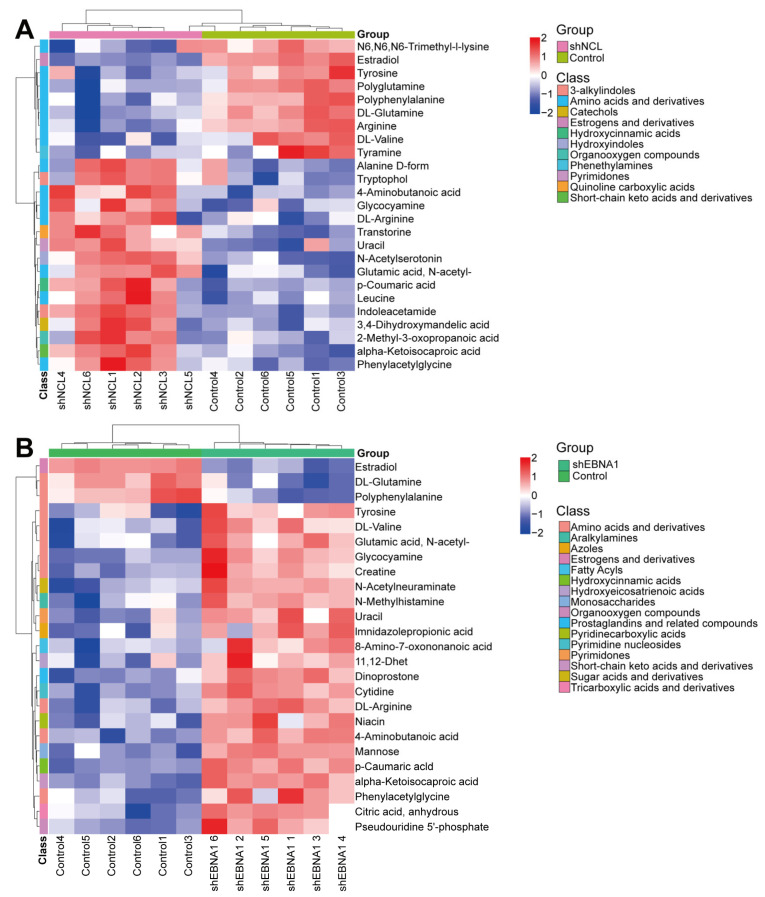
Hierarchical clustering heatmap of differential metabolites. (**A**) The group of “shNCL vs. Control”. (**B**) The group of “shEBNA1 vs. Control”.

In addition, we classified the abovementioned pathways at the first level and found that they are mainly involved in environmental information processing, genetic information processing, human diseases, metabolism, and organismal systems’ regulation (Supplementary Figure S5A,B). Hierarchical clustering heatmap showed that these differential metabolites were mainly classified into amino acids and derivatives, estrogens and derivatives, hydroxycinnamic acids, organooxygen compounds, pyrimidones, and short- chain keto acids and derivatives six categories (Figure 7A,B). The levels of 4-Aminobutanoic acid, alpha-Ketoisocaproic acid, DL-Arginine, Glutamic acid, N-acetyl-, Glycocyamine, p-Coumaric acid, phenylacetylglycine and uracil were significantly increased, while the levels of DL-Glutamine, estradiol, and polyphenylalanine were significantly decreased in the shNCL and shEBNA1 groups compared to the control group.

**Figure 8:**
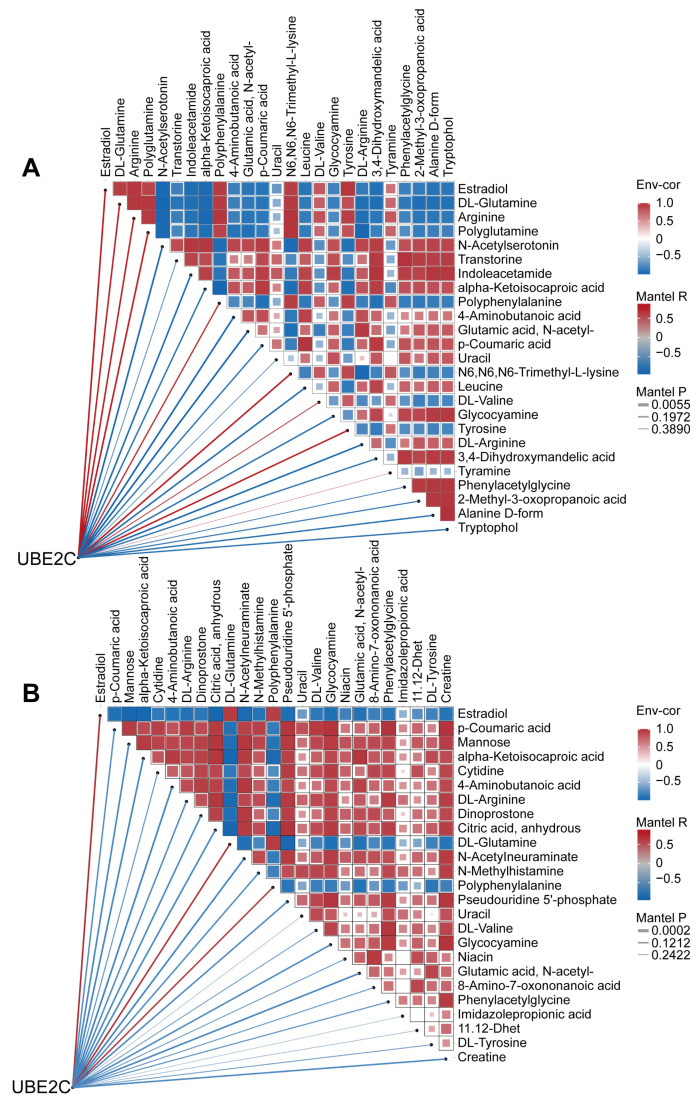
Combined metabolomics and proteomics analyses revealed that 4-Aminobutanoic acid (GABA) was highly negatively correlated with UBE2C. (**A**) Heatmap of the correlation network of UBE2C, NCL and differential metabolites in “shNCL vs. Control”. (**B**) Heatmap of correlation network of UBE2C, NCL with differential metabolites in “shEBNA1 vs. Control”.

Next, we performed correlation analyses of differential metabolites and UBE2C. The results showed that in the “shNCL vs. Control”, all differential metabolites and UBE2C showed high correlation (|R| > 0.6) except uracil and tyramine (Figure 8A). In the “shEBNA1 vs. Control”, except for uracil, imidazolepropionic acid and 11,12-Dhet, all other differential metabolites and UBE2C showed higher correlation (|R| > 0.6) (Figure 8B). Notably, 4-Aminobutanoic acid showed the strongest negative correlation with UBE2C: R = −0.9268, *P* = 0.0078 in the “shNCL vs. Control”, and R = −0.9885, *P* = 0.0002 in the “shEBNA1 vs. Control”. Therefore, we speculated that 4-Aminobutanoic acid played a key role in the NCL/EBNA1/UBE2C signaling pathway and might be a key regulatory molecule in curcumol inhibition of NPC invasion and migration.

### 3.5. Curcumol, shNCL, and shEBNA1 All Inhibited the Expression of UBE2C in EBV-Positive NPC In Vivo

**Figure 9:**
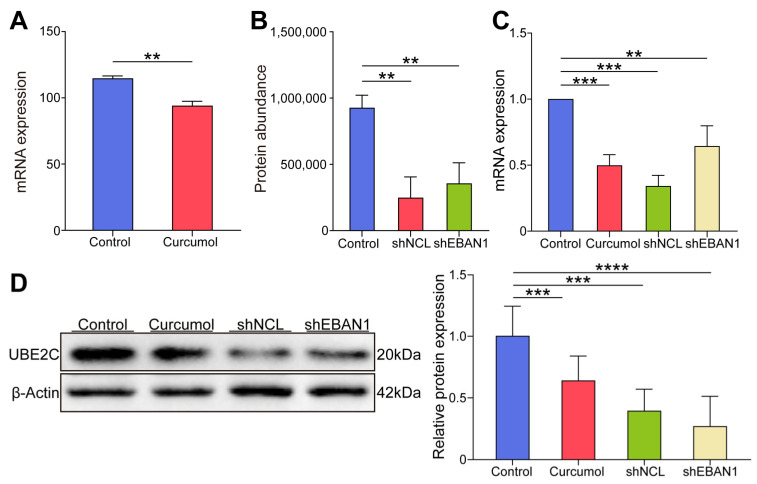
The expression of UBE2C in the experimental model of EBV-positive NPC both in vitro and in vivo. (**A**) mRNA expression levels of UBE2C in RNA-seq of NPC cells. (**B**) Protein abundance levels of UBE2C in proteomics of NPC cells. (**C**) qRT-PCR to detect the expression of UBE2C in the subcutaneously transplanted tumor tissue of nude mice. (**D**) WB to detect the expression of UBE2C in the subcutaneously transplanted tumor tissue of nude mice (data are presented as mean ± SEM. *n* = 3, ** *p*  <  0.01, *** *p*  <  0.001, **** *p*  <  0.0001). Original images can be found in Figure S7.

Among these common targets, we further focused on UBE2C, a key target on the ubiquitin-mediated proteolysis signaling pathway. To evaluate the expressed level of this critical target, the gene expression level and protein abundance of UBE2C were calculated; the findings indicated that UBE2C was highly expressed in the control group and significantly decreased after curcumol administration, and significantly decreased after knockdown of NCL or EBNA1 (Figure 9A,B). To evaluate the role of UBE2C in NCL/EBNA1-mediated curcumol inhibition of NPC invasion and migration, first, we established a nude mouse xenograft tumor model and randomly divided the mice into control, curcumol, shNCL, and shEBNA1 groups (referring to our previous study, the dosage of curcumol was 80 mg/kg once a day) [7]. Then, qRT-PCR and Western blotting were used to detect the expression of UBE2C in xenograft tumor tissues, respectively. When compared with the control group, the curcumol group, shNCL group, and shEBNA1 group all significantly reduced the mRNA expression level and protein expression level of UBE2C (Figure 9C,D). The findings indicated that curcumol suppressed EBV-positive NPC UBE2C expression both in vitro and in vivo, whereas NCL/EBNA1 promoted UBE2C expression.

### 3.6. Si-UBE2C Inhibited the Invasion and Migration of EBV-Positive NPC In Vitro

**Figure 10:**
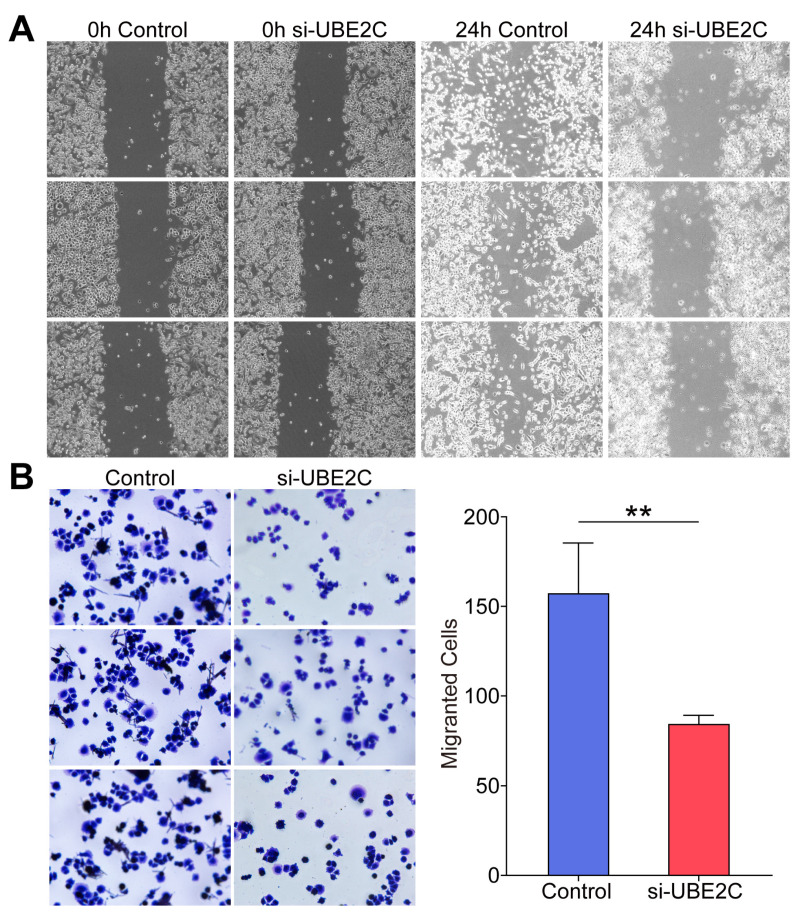
si-UBE2C inhibited EBV-positive NPC cell invasion and migration. (**A**) The migration ability of the C666-1 cell line after silencing UBE2C was detected by scratch assay. (**B**) The invasion ability of the C666-1 cell line after silencing of UBE2C was detected by transwell assay (data are presented as mean ± SEM. *n* = 3, ** *p*  <  0.01).

To investigate the specific role of UBE2C in the invasion and migration ability of EBV-positive NPC cells in depth, we constructed the C666-1 cell line in which UBE2C gene expression was silenced. Subsequently, the migratory and invasive properties of these cells were evaluated in the in vitro environment using the scratch assay as well as the transwell assays. The experimental results showed that after 24 h of scratch treatment, the migratory ability of UBE2C-silenced NPC cell lines was significantly reduced compared to the control group (Figure 10A), suggesting that UBE2C inhibition has a direct negative regulatory effect on cell migration. Similarly, the invasion ability of UBE2C-silenced NPC cell lines was significantly weaker than that of the control group (Figure 10B). In summary, the migration and invasion ability of EBV-positive NPC cells in vitro were effectively suppressed by inhibiting the expression of UBE2C.

## 4. Discussion

A malignant tumor, NPC occurs mainly in southern China. Although the 5-year survival rate has been improved after years of efforts, metastasis is still the main factor for recurrence and death [18]. Previous reports have demonstrated that curcumol has a significant anti-tumor effect on NPC. Further research found that curcumol inhibited the invasion and migration of NPC cells by targeting NCL and inhibiting EBNA1. To date, no transcriptomics or proteomics analyses of curcumol-treated NPC cells have been reported. Based on the above reasons, we used C666-1 cells (EBV-positive NPC cells) to establish the model, and then carried out treatment with curcumol, followed by transcriptomics analysis to explore the inhibitory mechanism of curcumol on NPC. GO annotation significantly enriched the regulation of protein ubiquitination, negative regulation of cell cycle, regulation of apoptotic signaling pathways, etc., indicating that these biological processes were likely to be related to the inhibition of NPC by curcumol. In addition, the CC and MF were also significantly enriched in ubiquitin ligase complex, ubiquitin-like protein ligase binding, cadherin binding, etc. KEGG pathway enrichment analysis showed that ubiquitin-mediated proteolysis, cell cycle, autophagy, apoptosis, and HIF-1 signaling pathways were related to tumor cell invasion and migration. Interestingly, the ubiquitin-mediated proteolysis occurred most frequently, indicating that this pathway plays an important role in the inhibition of NPC by curcumol.

Given previous reports on the important roles of NCL and EBNA1 in NPC invasion and migration, there is no proteomics analysis of shNCL or shEBNA1 in NPC. We knocked down NCL and EBNA1 and then performed proteomics analysis to explore how they affect the mechanism of action of NPC from the perspective of protein expression. The results showed that the GO annotations significantly enriched similarly after the two key proteins were knocked down separately, and the KEGG enrichment pathways analyses also enriched the ubiquitin-mediated proteolysis. Moreover, based on the synergistic effect of NCL and EBNA1, 39 up-regulated proteins and 27 down-regulated proteins were obtained by the intersection of the proteomics data. Then, combined with transcriptomics data analysis, there were nine up-regulated and six down-regulated targets. And UBE2C, a key protein in the ubiquitin-mediated proteolysis signaling pathway, is among them [19]. The results showed that UBE2C is a key protein in curcumol inhibiting the invasion and migration of NPC via NCL/EBNA1.

4-Aminobutanoic acid (GABA) is the primary inhibitory neurotransmitter in the mammalian central nervous system, playing a crucial role in reducing neuronal excitability [20]. Beyond its neurological functions, GABA has been implicated in various physiological processes, including immune regulation and cell proliferation [21]. In the context of cancer, GABA has been found to exhibit paradoxical roles, acting as both a tumor suppressor and a promoter, depending on the cellular context. For instance, high levels of GABA have been associated with increased tumorigenesis in some cancers, possibly through its effects on cell cycle regulation and apoptosis inhibition [22]. In NPC, GABA-induced apoptosis in tumor cells [23], and metabolomics data in this study indicate a significant dysregulation of GABA metabolism in response to NCL and EBNA1 knockdown, suggesting a potential link between GABA and the aggressive phenotype of NPC cells. While UBE2C is a key player in the ubiquitin-mediated proteolysis pathway, influencing cell cycle progression and apoptosis, GABA could potentially intersect with these processes through its effects on cellular metabolism and redox balance [24]. Given the established role of UBE2C in NPC and the novel finding of GABA dysregulation in our metabolomics study, it is plausible that UBE2C regulated the activity or expression of GABA directly or indirectly, thereby affecting the tumorigenic potential of NPC cells.

The results of proteomic and transcriptomic studies indicate that there is a significant correlation between nasopharyngeal carcinoma and ubiquitinated proteins, which indicates that UBE2C plays a key role in nasopharyngeal carcinoma. UBE2C, an important protein-coding gene, belongs to the E2 family of ubiquitin-conjugating enzymes. It participates in the ubiquitination process of substrate proteins by working closely with specific E3 enzymes, which ultimately degrades them [25,26,27]. In recent years, several studies have demonstrated that UBE2C exhibits high expression in a variety of malignant tumors [28,29,30]. This abnormally high expression demonstrates that UBE2C is not only a key player in the ubiquitin-mediated proteolysis pathway but also influences cell cycle progression and apoptosis, which is closely associated with poor patient prognosis [26,31,32]. UBE2C, through its function in promoting cell cycle progression, may exacerbate chromosomal instability, thereby contributing to tumor occurrence and progression [33]. The importance of UBE2C is growing, especially in the study of NPC. Since it was first reported in NPC in 2008 [34], subsequent studies have continued to reveal its key role in the disease. UBE2C was found to be highly expressed in NPC tissues and was strongly correlated with the degree of malignancy of the tumor [35]. Recent studies have even shown that the silencing of PARP-1-binding proteins by inhibiting UBE2C inhibits the invasion and migration of NPC cells [36].

**Figure 11:**
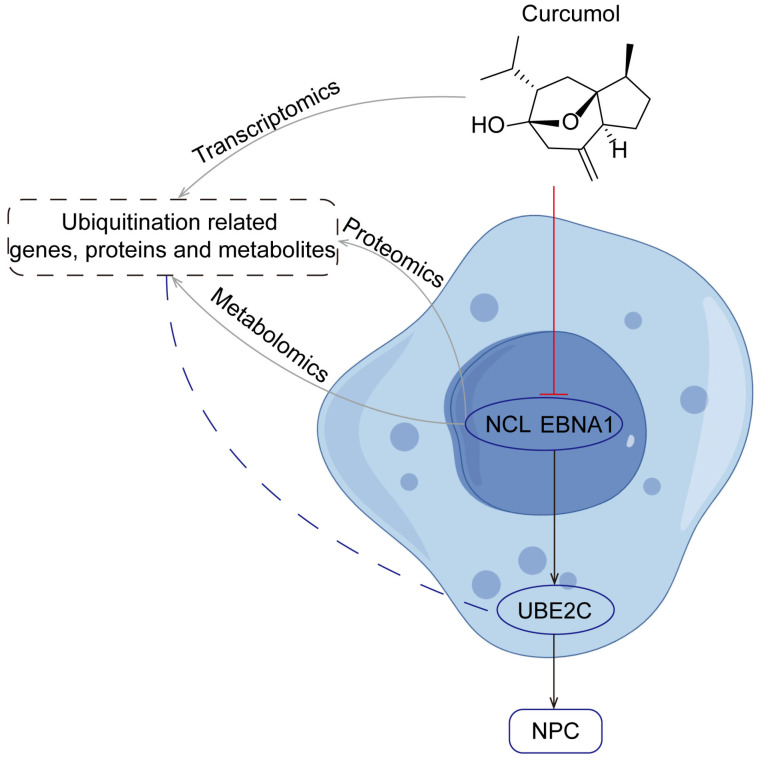
Mechanism of curcumol reducing NPC invasion and metastasis through inhibition of NCL/EBNA1/UBE2C explored by multi-omics.

In this study, UBE2C, as a downstream gene of NCL/EBNA1, played a central role in the invasion and metastasis of nasopharyngeal carcinoma. By down-regulating the expression of the NCL/EBNA1/UBE2C axis using curcumol, the invasion and migration of NPC cells can be effectively inhibited, providing a new strategy and direction for the treatment of NPC (Figure 11). However, as a ubiquitination protein, how UBE2C functions to cause protein degradation in the abovementioned processes needs to be further researched. The present study is not only highly original, but also closely related to NPC pathogenesis, therapeutic strategies and drug development, and has important implications in the field of NPC.

## 5. Conclusions

Curcumol, as a natural compound, significantly inhibited the invasion and migration ability of EBV-positive NPC cells by effectively down-regulating the expression levels of NCL/EBNA1 and UBE2C in vitro experiments and in vivo models. This finding not only revealed a potential new pathway of curcumol in NPC treatment but also opened up important ideas for the development of novel therapeutic regimens for NPC patients. It also enriched our understanding of the molecular mechanisms of how natural drugs exert their anti-tumor effects, provided valuable insights for the comprehensive elucidation of the pharmacological activities of these drugs, and further promoted their exploration and possibilities in clinical applications.

## Figures and Tables

**Table 1:** Sequences of forward and reverse primers used in qRT-PCR.

Name	Sequence(5′-3′)
UBE2C Forward	GAATCTCCGCCTTCCCTGAG
UBE2C Reverse	GGGTTTTTCCAGAGTTCCGC
NCL Forward	TGAACCAGCAGCGATGAAAG
NCL Reverse	CCACGTTCTTGGCTTTCACA
EBNA1 Forward	GGCAGTGGACCTCAAAGAAGAG
EBNA1 Reverse	CAATGCAACTTGGACGTTTTT
ACTIN Forward	AAAGACCTGTACGCCAACAC
ACTIN Reverse	GTCATACTCCTGCTTGCTGAT

## Data Availability

The original contributions presented in the study are included in the article; further inquiries can be directed to the author.

## Supplementary Materials

The following supporting information can be downloaded at: https://www.mdpi.com/article/10.3390/biom14091142/s1. Figure S1: GO analysis of DEPs in NPC cells with shNCL or shEBNA1, enriched GO terms in terms of BP, CC, and MF, respectively. Figure S2: Heatmaps of 66 common DEPs in the same direction each. Figure S3: Differential metabolite analysis of “shNCL vs Control”. Figure S4: Differential metabolite analysis of “shEBNA1 vs Control”. Figure S5: Screening of UBE2C-related metabolic pathways and differential metabolites. Figure S6: Screening of UBE2C-related metabolic pathways and differential metabolites. Figure S7: Original western blot images. Original images can be found in Figures S1–S7.
