# Supplementary material for: Multi-Omics Exploration of the Mechanism of Curcumol to Reduce Invasion and Metastasis of Nasopharyngeal Carcinoma by Inhibiting NCL/EBNA1-Mediated UBE2C Upregulation
Source: Biomolecules. 2024 Sep 9;14(9):1142. doi: 10.3390/biom14091142 (PMC11430640; doi:10.3390/biom14091142)
Supplement: Supplementary file 1 [file biomolecules-14-01142-s001.zip › biomolecules-3149571-supplementary.pdf]

Supplementary

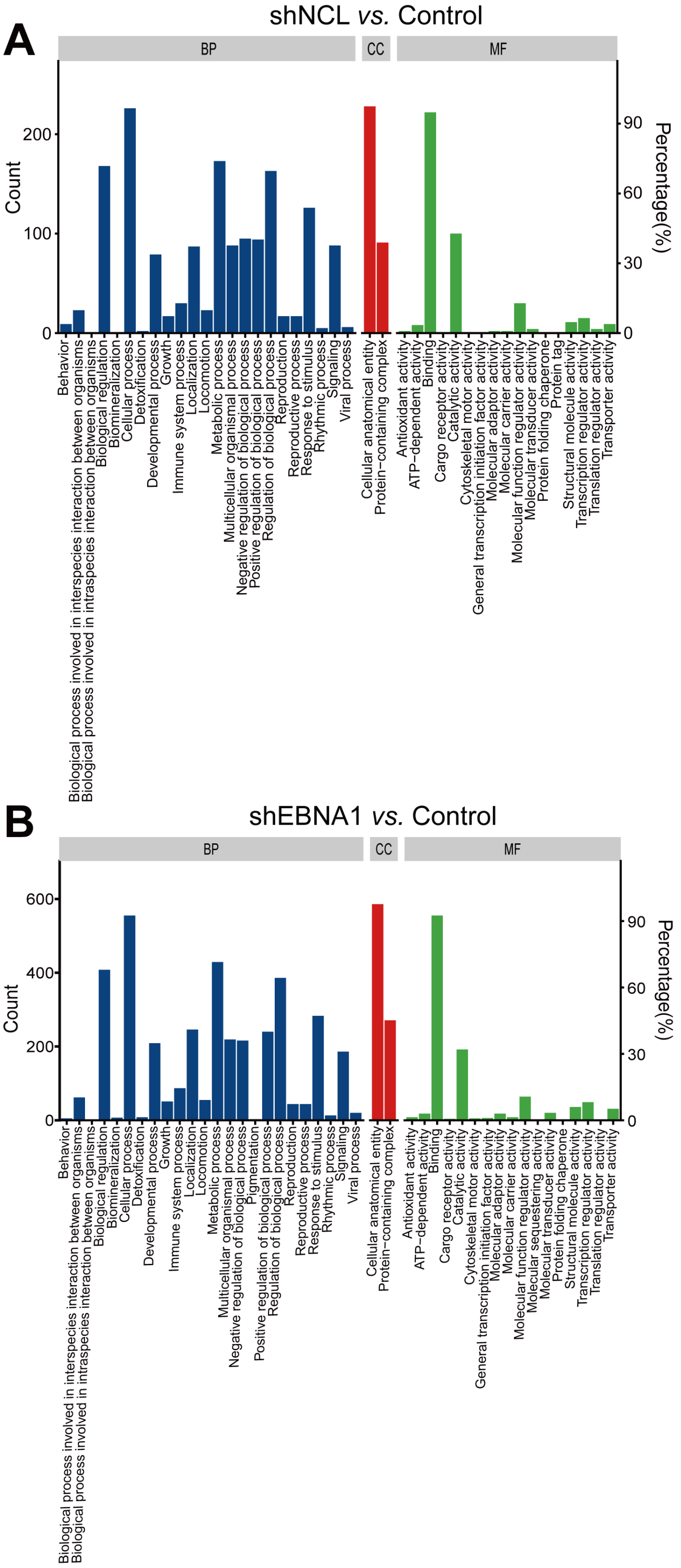

Figure S1. GO analysis of DEPs in NPC cells with shNCL or shEBNA1, enriched GO terms in terms of BP, CC, and MF, respectively. (**A**) The group of "shNCL vs Control". (**B**) The group of "shEBNA1 vs Control".

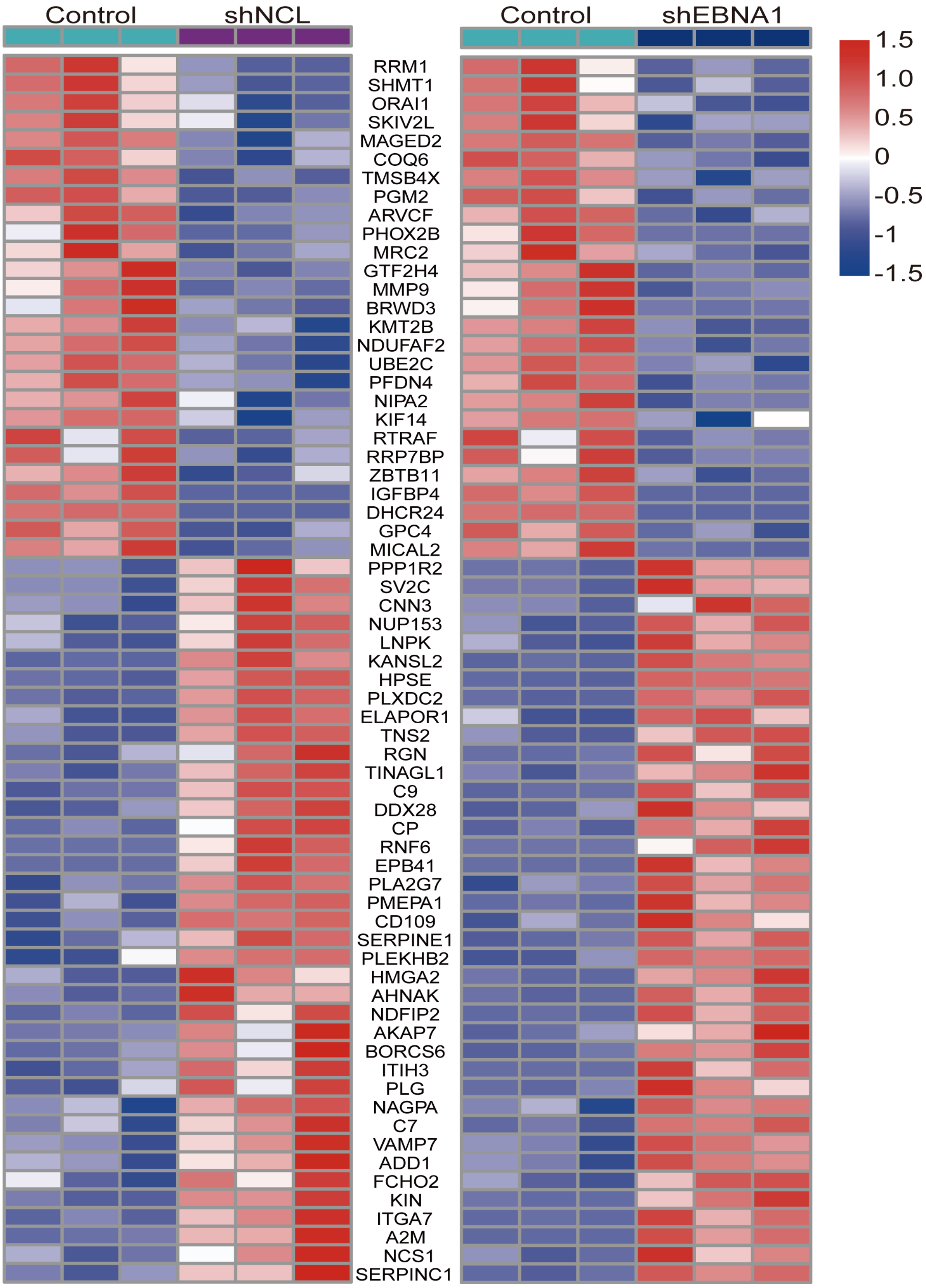

Figure S2. Heatmaps of 66 common DEPs in the same direction each.

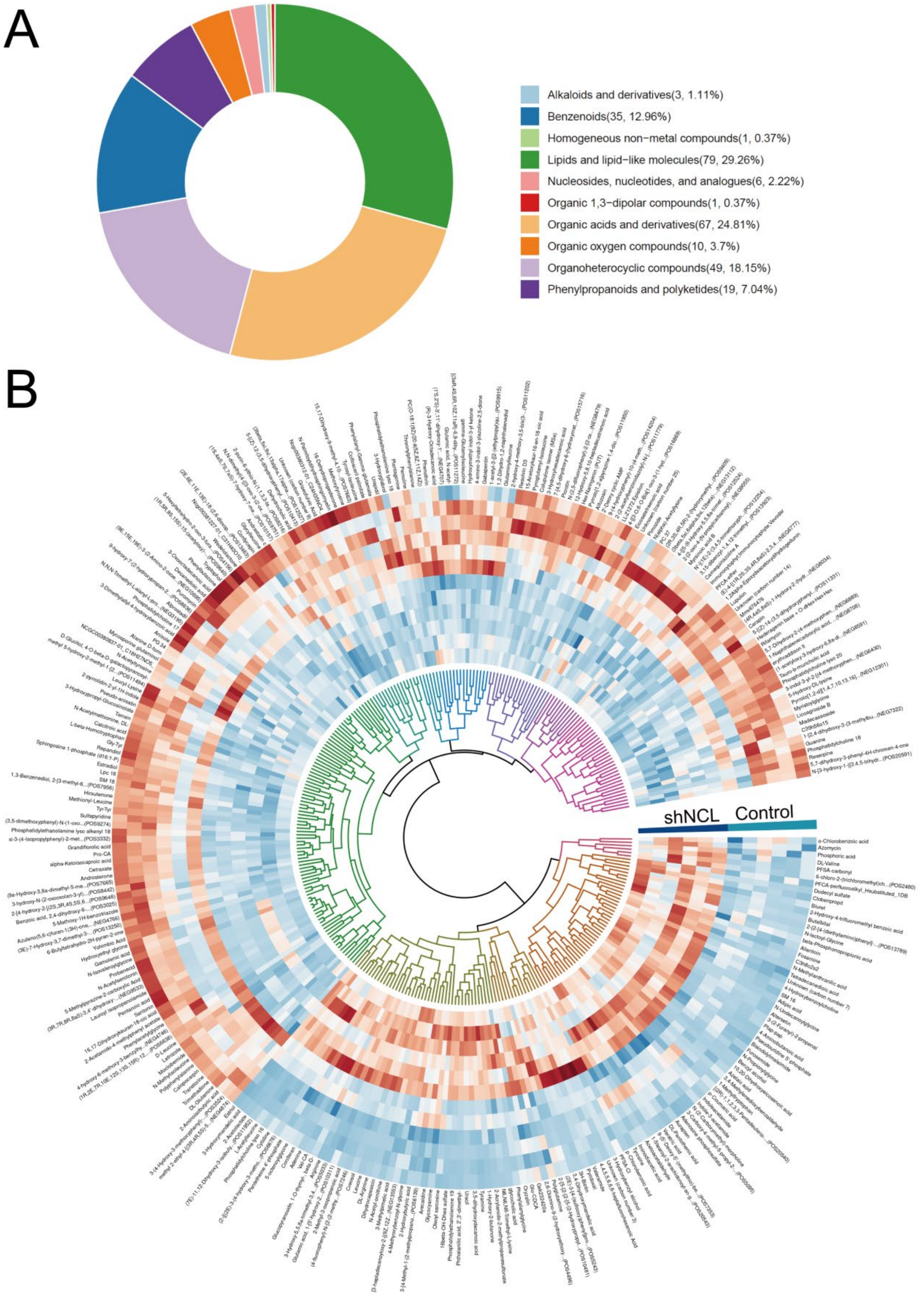

Figure S3. Differential metabolite analysis of "shNCL vs Control". **(A)** HMDB Super Class.**(B)** Circular Heatmap.

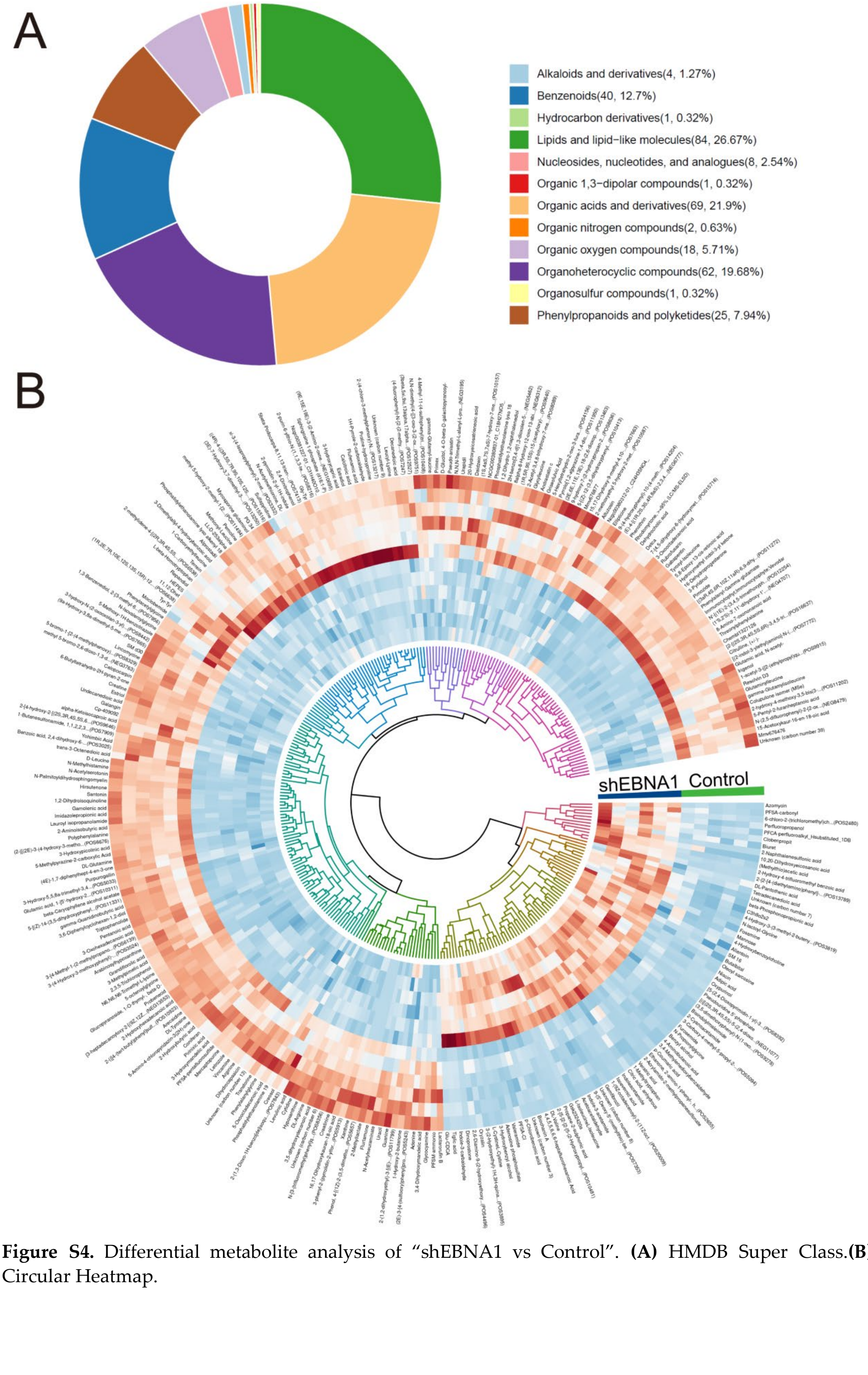

Figure S4. Differential metabolite analysis of "shEBNA1 vs Control". **(A)** HMDB Super Class. **(B)** Circular Heatmap.

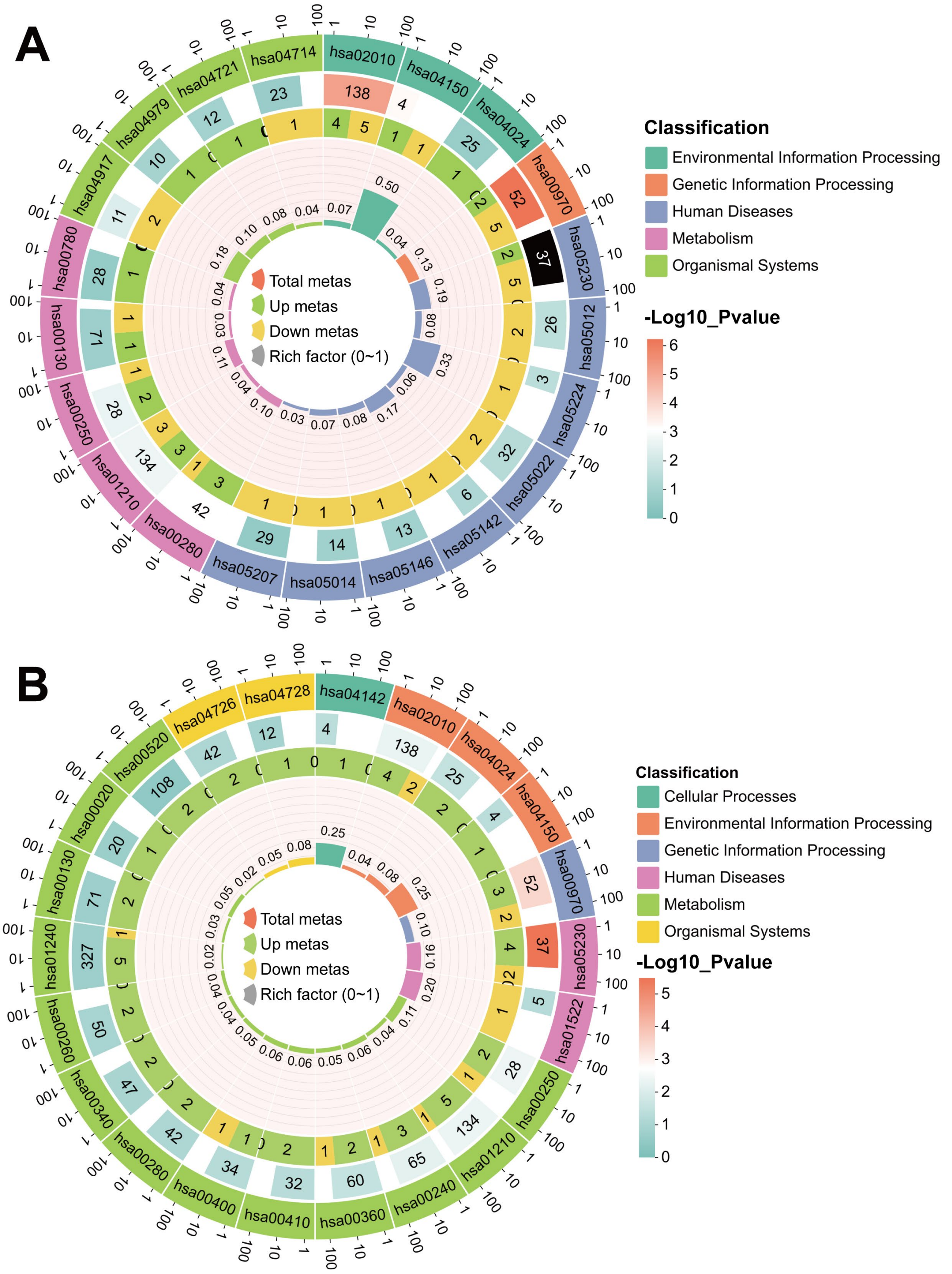

Figure S5. Screening of UBE2C-related metabolic pathways and differential metabolites. (**A**) KEGG circle plot of "shNCL vs Control". (**B**) KEGG circle plot of "shEBNA1 vs Control".

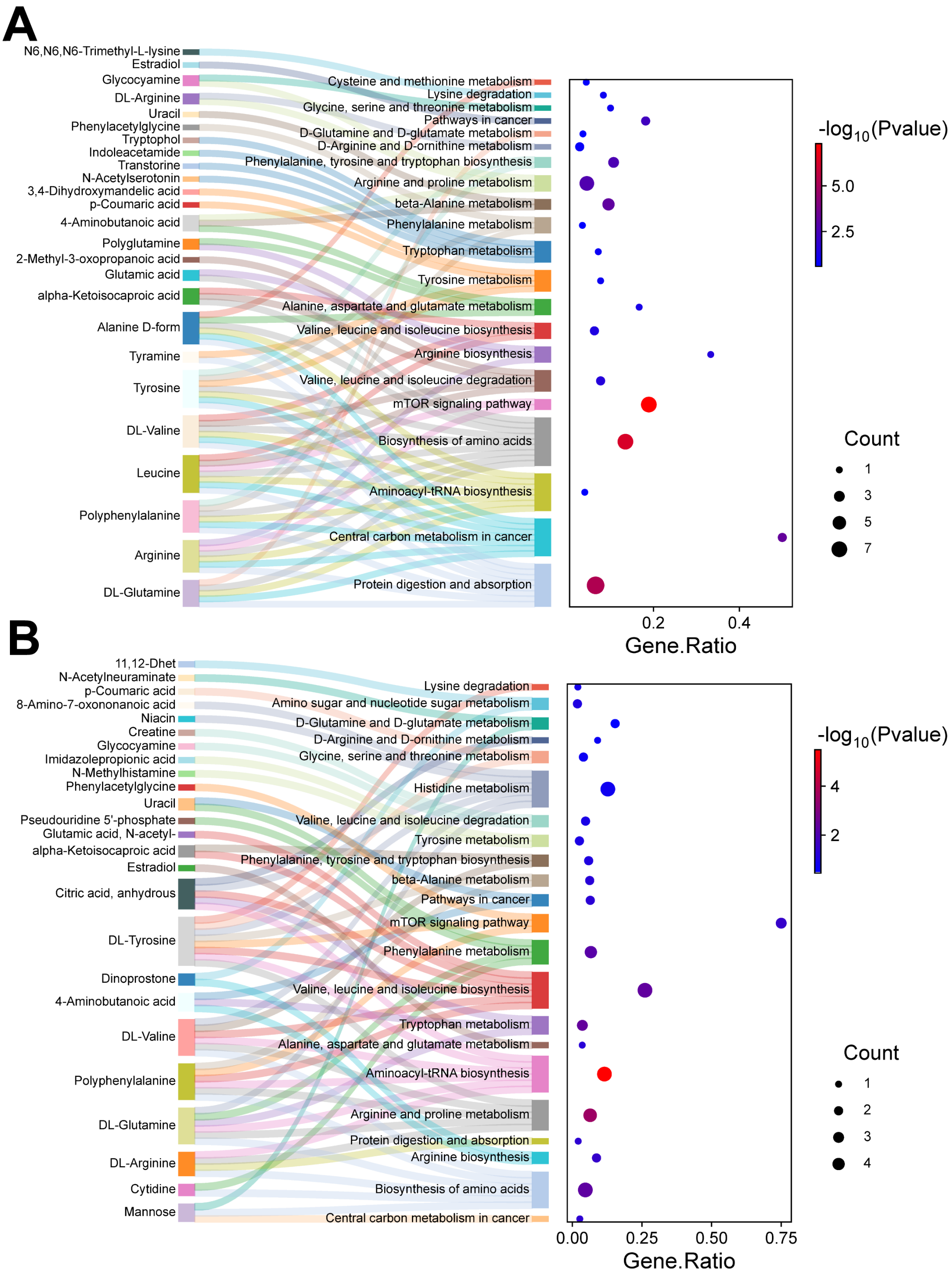

Figure S6. Screening of UBE2C-related metabolic pathways and differential metabolites. (**A**) KEGG Sankey bubble plots of “shNCL *vs* Control”. (**B**) KEGG Sankey bubble plots of “shEBNA1 vs Control”.

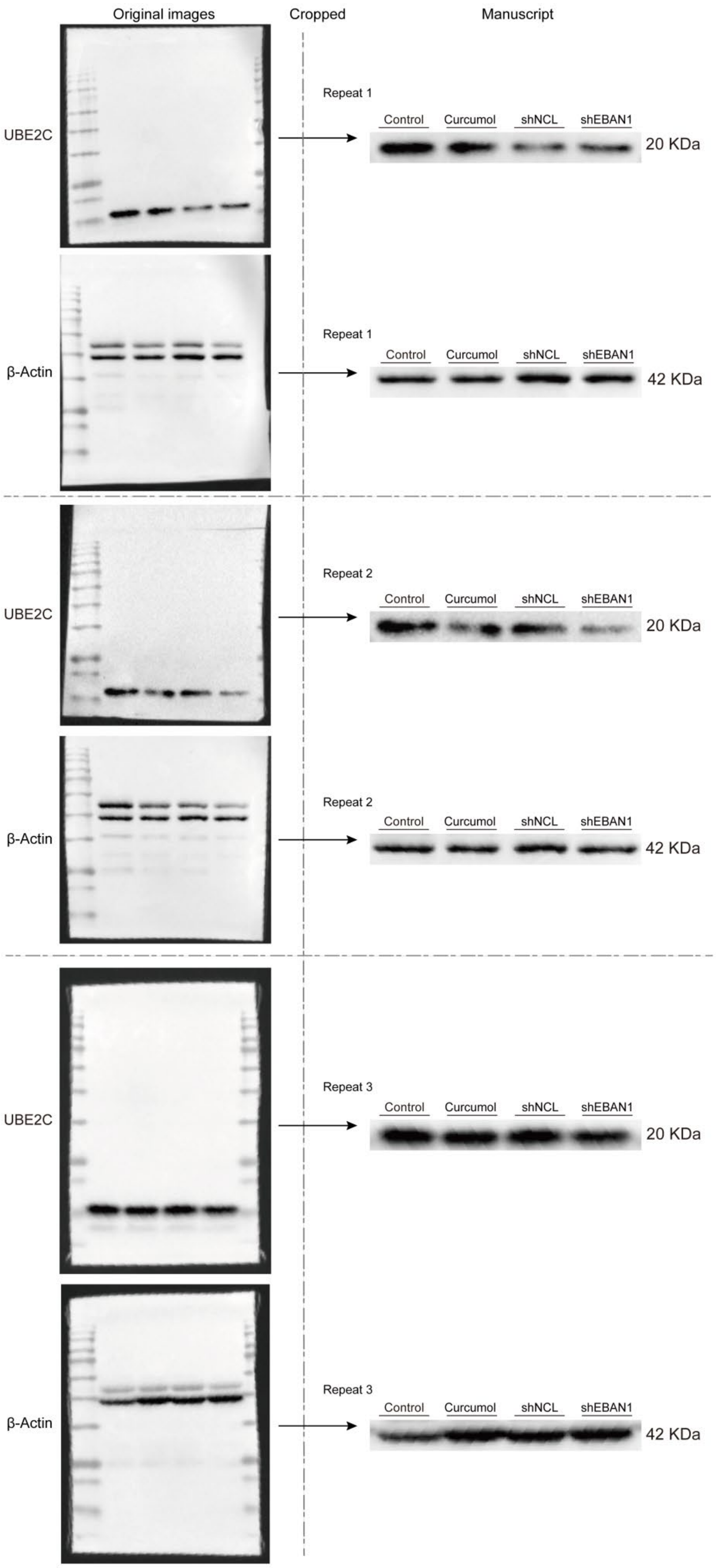

Figure S7. Original western blot images
